# CRISPR Interference of a Clonally Variant GC-Rich Noncoding RNA Family Leads to General Repression of *var* Genes in Plasmodium falciparum

**DOI:** 10.1128/mBio.03054-19

**Published:** 2020-01-21

**Authors:** Anna Barcons-Simon, Carlos Cordon-Obras, Julien Guizetti, Jessica M. Bryant, Artur Scherf

**Affiliations:** a Unité de Biologie des Interactions Hôte-Parasite, Institut Pasteur, Paris, France; b INSERM U1201, Paris, France; c CNRS ERL9195, Paris, France; d Sorbonne Université, Ecole doctorale Complexité du Vivant ED515, Paris, France; Washington University School of Medicine

**Keywords:** ncRNA, *Plasmodium falciparum*, antigenic variation, mutually exclusive expression, epigenetics, *var* genes, virulence, malaria parasites

## Abstract

The human malaria parasite Plasmodium falciparum uses mutually exclusive expression of the PfEMP1-encoding *var* gene family to evade the host immune system. Despite progress in the molecular understanding of the default silencing mechanism, the activation mechanism of the uniquely expressed *var* member remains elusive. A GC-rich noncoding RNA (ncRNA) gene family has coevolved with *Plasmodium* species that express *var* genes. Here, we show that this ncRNA family is transcribed in a clonally variant manner, with predominant transcription of a single member occurring when the ncRNA is located adjacent to and upstream of an active *var* gene. We developed a specific CRISPR interference (CRISPRi) strategy that allowed for the transcriptional repression of all GC-rich members. A lack of GC-rich ncRNA transcription led to the downregulation of the entire *var* gene family in ring-stage parasites. Strikingly, in mature blood-stage parasites, the GC-rich ncRNA CRISPRi affected the transcription patterns of other clonally variant gene families, including the downregulation of all *Pfmc-2TM* members. We provide evidence for the key role of GC-rich ncRNA transcription in *var* gene activation and discovered a molecular link between the transcriptional control of various clonally variant multigene families involved in parasite virulence. This work opens new avenues for elucidating the molecular processes that control immune evasion and pathogenesis in P. falciparum.

## Abstract (precis)

Plasmodium falciparum is the deadliest malaria parasite species, accounting for the vast majority of disease cases and deaths. The virulence of this parasite is reliant upon the mutually exclusive expression of cytoadherence proteins encoded by the 60-member *var* gene family. Antigenic variation of this multigene family serves as an immune evasion mechanism, ultimately leading to chronic infection and pathogenesis. Understanding the regulation mechanism of antigenic variation is key to developing new therapeutic and control strategies. Our study uncovers a novel layer in the epigenetic regulation of transcription of this family of virulence genes by means of a multigene-targeting CRISPR interference approach.

## INTRODUCTION

**FIG 1:**
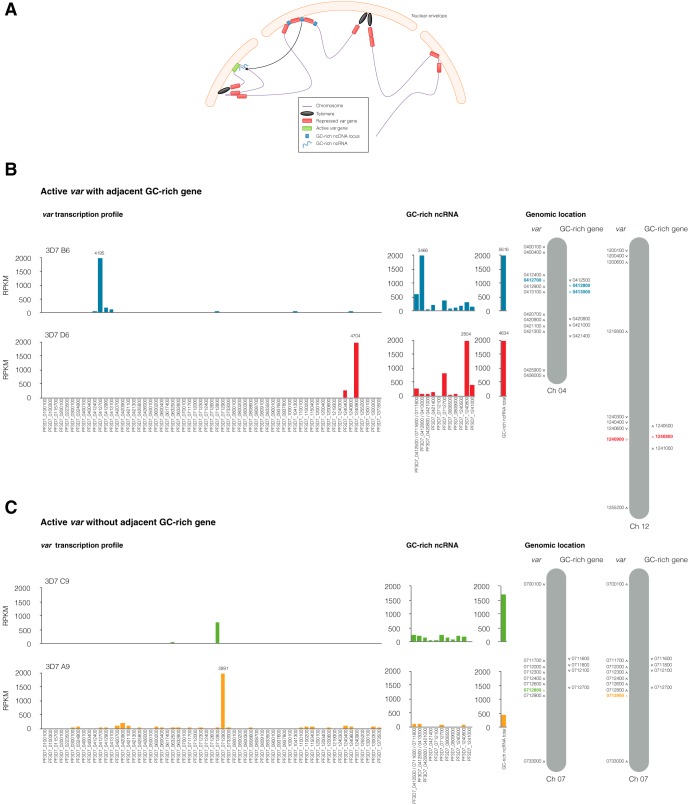
GC-rich ncRNA and *var* clonal variation. (A) Schematic model showing repressed *var* gene perinuclear clustering and active *var* gene relocation to the active expression site colocalizing with GC-rich ncRNA. ncDNA, noncoding DNA. (B) Transcriptional profile of *var* genes and GC-rich ncRNA at 12 hpi in WT clones assessed by RNA sequencing. Chromosome schematics (modified from reference 44) highlight the active *var* genes and GC-rich genes in different clones. Clones 3D7 B6 (blue) and 3D7 D6 (red) have predominant transcription of the GC-rich ncRNA adjacent to their active *var* locus. (C) Transcriptional profile of *var* genes and GC-rich ncRNA at 12 hpi in clones 3D7 C9 (green) and 3D7 A9 (yellow), which do not have a GC-rich gene upstream from their respective active central *var* loci and which have several GC-rich ncRNA transcripts at lower levels. The values are the means of the RPKM from two independent experiments at 12 hpi. The results for further clones are shown in Fig. S1 in the supplemental material.

The protozoan parasite Plasmodium falciparum is responsible for the deadliest form of human malaria, causing hundreds of thousands of deaths every year (1), and, akin to other protozoan parasites (2), relies on the mutually exclusive expression of virulence gene families to survive within the host (2–6). Among them, the 60-member *var* gene family encodes PfEMP1, an important variant surface adhesion molecule (7). *var* genes are located in subtelomeric regions of 13 out of 14 chromosomes and in central clusters of chromosomes 4, 6, 7, 8, and 12 (8), and their transcription is epigenetically controlled (4). Transcription of a single *var* gene peaks at about 12 h post invasion (hpi) (9) and is then silenced but poised during the later stages of the 48-h intraerythrocytic cycle (10, 11). All other *var* genes remain transcriptionally silenced throughout the cycle and are tethered in repressive heterochromatic clusters enriched in H3K9me3 and H3K36me3 at the nuclear periphery (10, 12, 13). In contrast, the active *var* gene is euchromatic, enriched in H3K4me3 and H3K9ac, and localizes to a distinct perinuclear expression site (10, 12, 14) (a schematic is shown in Fig. 1A). Since the first description of this expression site (14), the mechanism of activation has remained elusive. Long noncoding RNAs (lncRNA) transcribed from the *var* intron have been implicated in a *cis* activation process (15, 16); however, the necessity of the intron-derived lncRNA was questioned by a recent study showing that an intron-less *var* gene could be activated and silenced (17).

10.1128/mBio.03054-19.1 FIG S1 GC-rich ncRNA and *var* clonal variation. The transcriptional profiles of *var* genes and GC-rich ncRNA at 12 hpi in WT clones, assessed by RNA sequencing, are shown. Chromosome schematics (modified from reference 44) highlight active *var* genes and GC-rich genes in different clones. (A) Clone 3D7 A6 (red) has predominant transcription from the GC-rich gene (PF3D7_0808500) adjacent to the active *var* locus (PF3D7_0808600). (B) Clone 3D7 B9 (green) has two dominant active *var* genes, one central and one subtelomeric, and several GC-rich ncRNA transcripts, with the one adjacent to the active central *var* being dominant. CSA-panned clone 3D7 (brown) has predominant transcription from subtelomeric *var2csa* and several GC-rich ncRNA transcripts. The data are the mean RPKM from two independent experiments at 12 hpi. Download FIG S1, PDF file, 0.1 MB. Copyright © 2020 Barcons-Simon et al. 2020 Barcons-Simon et al. This content is distributed under the terms of the Creative Commons Attribution 4.0 International license.

The initial characterization of a gene family encoding 15 GC-rich noncoding RNAs (annotated *RUF6*) located adjacent to central *var* genes suggested a role in the regulation of *var* genes (18, 19). Fluorescence *in situ* hybridization (FISH) revealed that these ncRNAs colocalized to the *var* gene expression site and that episomal overexpression of distinct GC-rich ncRNAs resulted in the deregulation of mutually exclusive *var* gene expression (18). However, their mechanism of action remains unknown. In this study, we used CRISPR interference (CRISPRi) to downregulate the entire GC-rich gene family and provide evidence for the necessity of the GC-rich ncRNA in mutually exclusive *var* gene activation. Our results demonstrate a clear link between the transcription of both gene families, along with other clonally variant gene families involved in malaria parasite virulence.

## RESULTS

### GC-rich ncRNA shows predominant transcription of a single member when adjacent to an active *var* gene.

Recently, RNA FISH was used to demonstrate the physical association of GC-rich ncRNAs with the expression site of central and subtelomeric *var* genes (18). Given the restricted genomic location of the GC-rich genes adjacent to some, but not all, central *var* genes (Fig. 1A), we investigated whether the transcription of GC-rich and *var* multigene families is coordinated. To this end, we generated an array of P. falciparum 3D7 wild-type (WT) clones and performed paired-end RNA sequencing (RNA-seq) analysis at 12 hpi to determine the transcriptional profile of the highly homologous GC-rich ncRNA and *var* gene families. In three clones, a single member of the 15 GC-rich genes was predominantly transcribed (Fig. 1B; see also Fig. S1A in the supplemental material). In the same clones, we observed mutually exclusive transcription of the central *var* gene adjacent to the upstream region of the active GC-rich gene (Fig. 1B and Fig. S1A). Notably, when the active central *var* gene lacks an adjacent GC-rich gene, ncRNA transcripts from several loci were detected, but at levels much lower than those in the former situation (Fig. 1C). Since subtelomeric *var* genes are prone to switch faster in culture, we were able to isolate only a clone with a dominant subtelomeric *var* gene that also expressed a second central *var* gene. In this clone, we observed low-level transcription from several GC-rich ncRNA genes, in addition to the dominant GC-rich ncRNA adjacent to the transcribed central *var* (Fig. S1B). Additionally, we performed receptor panning with chondroitin sulfate A (CSA) to enrich for parasites expressing a single subtelomeric member called *var2csa*. In this parasite panned line, we observed predominant transcription of *var2csa* and low levels of transcripts from several GC-rich genes (Fig. S1B). Taken together, these data suggest that the GC-rich gene family is expressed in a clonally variant manner related to the chromosomal location of the active *var* gene. GC-rich genes are predominantly transcribed from a single locus when adjacent to and upstream of an active central *var* gene.

### CRISPRi of the entire GC-rich gene family leads to transcriptional downregulation of the *var* gene family.

**FIG 2:**
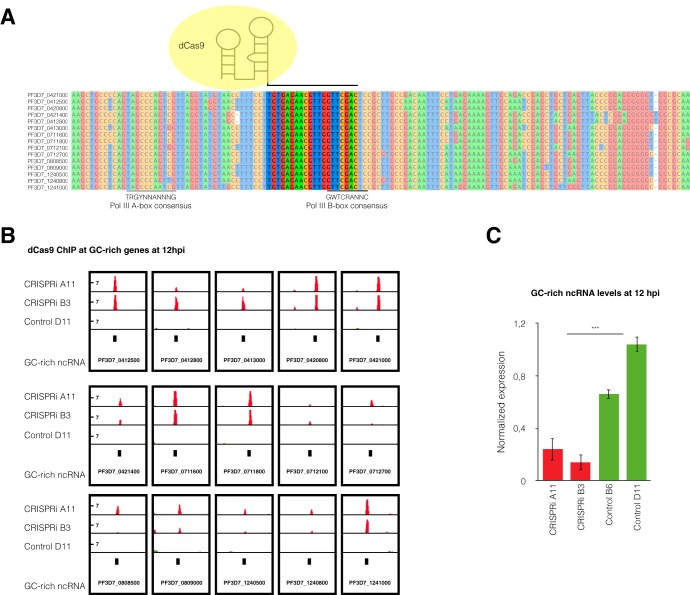
GC-rich ncRNA knockdown by CRISPR interference. (A) Multiple-sequence alignment of the 15 GC-rich ncRNA members showing the dCas9 target region (not shaded), where the sgRNA binds to the DNA coding strand of all GC-rich genes. The black lines at the bottom show the positions of the polymerase III A- and B-box consensus motifs (45). (B) ChIP sequencing data show the enrichment of dCas9 in the 15 GC-rich gene loci for the CRISPRi line. The logarithmic scale of the likelihood ratio of the fold enrichment over the input level for dCas9 computed with MACS2 software is represented in red for the CRISPRi clones and in green for scrambled control clone D11. The data range for each track is 0 to 14. Data are representative of two independent experiments at 12 hpi. (C) GC-rich ncRNA levels at 12 hpi quantified by RT-qPCR for two GC-rich gene CRISPRi clones (B3 and A11) and two scrambled control clones (control D11 and B6). The levels of expression are normalized to the fructose-bisphosphate aldolase (PF3D7_1444800) transcription levels. The means ± SEMs from three independent experiments are shown. Statistical significance was determined by two-tailed Student’s *t* test. ***, *P* < 0.001.

To determine the role of GC-rich ncRNA in *var* gene expression regulation, we aimed to downregulate the transcription of all GC-rich ncRNA genes. An attempted simultaneous knockout of all 15 highly homologous members was unsuccessful, likely due to the widespread distribution of the GC-rich genes or the diversity of their up- and downstream regions required for such an approach. Thus, we developed a CRISPR interference (CRISPRi) (20) strategy for multigene families to block transcription via binding of dead Cas9 (dCas9), a mutated Cas9 protein lacking endonuclease activity. We designed a single guide RNA (sgRNA) targeting a homologous region common to all GC-rich gene members (Fig. 2A). We transfected the 3D7 G7 parasite strain (a WT clone expressing the central *var* gene PF3D7_0412700) with the pUF-dCas9-GFP-3HA plasmid and either pL8-gRNA-GC-tc or a control plasmid with a scrambled guide RNA (pL8-gRNA-control). Chromatin immunoprecipitation (ChIP) of dCas9 followed by quantitative PCR (qPCR) with universal GC-rich gene primers showed a strong enrichment of dCas9 at GC-rich ncRNA loci in two independent CRISPRi clones but not in two scrambled control clones (Fig. S2A). ChIP followed by massively parallel DNA sequencing (ChIP-seq) analysis of dCas9 showed enrichment at all 15 GC-rich gene loci in both CRISPRi clones but not in the scrambled control (at 12 hpi in Fig. 2B and 24 hpi in Fig. S2B).

10.1128/mBio.03054-19.2 FIG S2 GC-rich ncRNA knockdown by CRISPR interference. (A) dCas9 enrichment at GC-rich genes calculated as a percentage of the input amount quantified by qPCR for two GC-rich ncRNA knockdown clones (CRISPRi B3 and A11) and two control clones with dCas9 and a control gRNA (control D11 and B6) at 12 hpi. (B) ChIP sequencing data show the enrichment of dCas9 in the 15 GC-rich gene loci for the CRISPRi line. The logarithmic scale of the likelihood ratio of the fold enrichment over the input level for dCas9 computed with MACS2 software is represented in red for the CRISPRi clones and in green for the control gRNA clone D11. The data range for each track is 0 to 20. Data are representative of two independent experiments at 24 hpi. Download FIG S2, PDF file, 0.6 MB. Copyright © 2020 Barcons-Simon et al. 2020 Barcons-Simon et al. This content is distributed under the terms of the Creative Commons Attribution 4.0 International license.

To determine the transcriptional effect of GC-rich gene CRISPRi, we performed reverse transcription-quantitative PCR (RT-qPCR) with universal GC-rich ncRNA primers for two clones each of the CRISPRi and scrambled control lines. The housekeeping gene fructose-bisphosphate aldolase (PF3D7_1444800) was used for normalization. CRISPRi clones showed significantly reduced levels of GC-rich ncRNA compared to the control line clones at 12 and 24 hpi (see Fig. 2C and 4A, respectively).

**FIG 3:**
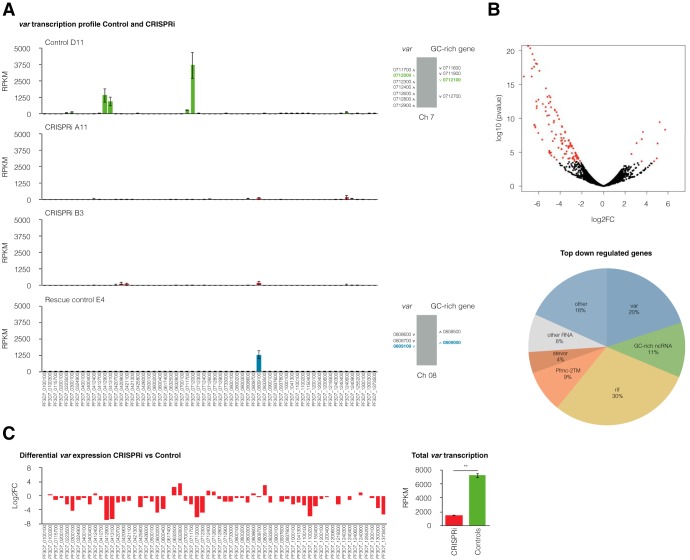
GC-rich ncRNA knockdown leads to the downregulation of *var* gene expression. (A) (Left) Transcriptional *var* gene profile at 12 hpi assessed by RNA sequencing for the control gRNA clone D11, two GC-rich ncRNA knockdown clones (CRISPRi B3 and A11), and the rescue control (clone E4). Two control gRNA clones and two rescue control clones were analyzed, but only one representative example for each control is shown. The means ± SEMs from three independent experiments are shown. (Right) Chromosome cluster schematics highlighting active *var* and GC-rich genes in control clones. (B) (Top) Volcano plot for differential expression between CRISPRi clones and the scrambled control line. Differentially expressed genes with a 0.01 FDR cutoff are represented by red dots. The total number of significantly differentially expressed genes was 125, 92% of which were downregulated and 8% of which were upregulated. (Bottom) Families of top downregulated genes in CRISPRi clones compared to scrambled control clones, significantly differentially expressed with a 0.01 FDR cutoff in three independent replicates. (C) (Left) Differential expression of *var* genes for CRISPRi clones compared to the scrambled control line shows the downregulation of the entire *var* gene family. The log of the fold enrichment (log_2_ fold change [log2FC]) for three replicates of two CRISPRi clones compared to two scrambled control clones is represented. (Right) Mean ± SEM transcriptional levels of the *var* gene family, as assessed by RNA-seq, for three replicates of two CRISPRi and two scrambled control clones. Statistical significance was determined by two-tailed Student’s *t* test. **, *P* < 0.01.

The global transcriptional effects of GC-rich gene CRISPRi were analyzed by RNA-seq. Strikingly, two independent clones of the CRISPRi lines exhibited a global downregulation of *var* genes, suggesting a role for the GC-rich ncRNA in the activation of *var* gene transcription (Fig. 3A). Conversely, scrambled control clones showed transcription of a single dominant *var* gene (Fig. 3A), similar to wild-type clones (Fig. 1B), suggesting that the expression of dCas9 alone does not affect *var* gene transcription. Additionally, a rescue experiment was conducted by removing the drug pressure required to maintain the plasmids and using negative drug selection to ensure plasmid removal from the CRISPRi lines (Fig. S3). Rescue control clones recovered *var* mutually exclusive transcription (Fig. 3A). Differential gene expression analysis of CRISPRi and scrambled control clones with a false discovery rate (FDR) cutoff of 0.01 returned 125 genes, 115 (92%) of which were downregulated in the CRISPRi lines in three independent replicates (Fig. 3B and C, Fig. S4, and Table S1). Among these downregulated genes were 13 GC-rich genes, 23 *var* genes (including the active *var* gene in the scrambled control clones, PF3D7_0712000), and several *rif* genes (Table S1). Differentially expressed genes other than GC-rich genes were validated for the lack of presentation of off-target dCas9 binding (Table S2). Transfection of CRISPRi plasmids into CSA-panned parasites also caused the line to display a downregulation of the GC-rich ncRNA and the repression of *var* mutually exclusive expression (Fig. S5). Altogether, our results suggest that GC-rich ncRNA transcription is essential for the mutually exclusive expression of *var* genes.

10.1128/mBio.03054-19.3 FIG S3 CRISPRi rescue phenotype experiment. PCR results with oligonucleotides for the dihydrofolate reductase (DHFR) cassette from pL8 plasmids on parasite gDNA. The plasmid was successfully removed from all rescue control clones (first 9 lanes). Plasmid removal was obtained by removing drug pressure and using negative selection with 5-fluorocytosine (anc.) over 21 days. Subsequently, clones were obtained by limiting dilution. The pL8 plasmid and gDNA from a WT strain were used as controls. Download FIG S3, PDF file, 0.03 MB. Copyright © 2020 Barcons-Simon et al. 2020 Barcons-Simon et al. This content is distributed under the terms of the Creative Commons Attribution 4.0 International license.

10.1128/mBio.03054-19.4 FIG S4 Differential gene expression in GC-rich ncRNA knockdown at 12 hpi. (A) Mean-variance scatterplot for differential expression between CRISPRi clones (A11 and B3) and scrambled control clones (D11 and B6) for three independent replicates at 12 hpi. (B) Volcano plot for differential expression between CRISPRi clones (A11 and B3) and scrambled control clones (D11 and B6) for three independent replicates at 12 hpi. Differentially expressed genes with a 0.01 FDR cutoff are represented by red dots. (C) Plot showing the percentage of up- and downregulated genes among the differentially expressed genes with a 0.01 FDR cutoff in CRISPRi clones (A11 and B3) compared to their expression in the scrambled control clones (D11 and B6) for three independent replicates at 12 hpi. (D) Families of top downregulated genes in CRISPRi clones (A11 and B3) significantly differentially expressed with a 0.01 FDR cutoff compared to their expression in the scrambled control clones (D11 and B6) in three independent replicates at 12 hpi. Download FIG S4, PDF file, 1.3 MB. Copyright © 2020 Barcons-Simon et al. 2020 Barcons-Simon et al. This content is distributed under the terms of the Creative Commons Attribution 4.0 International license.

10.1128/mBio.03054-19.5 FIG S5 GC-rich ncRNA knockdown by CRISPR interference on CSA-panned parasites. (A) GC-rich ncRNA levels at 12 hpi, as quantified by RT-qPCR, for two CRISPRi clones (D10 and J2) of a transfection on CSA-panned parasites and two control gRNA clones (control D11 and B6). The level of transcription was normalized to housekeeping gene fructose-bisphosphate aldolase (PF3D7_1444800) levels. The means ± SEMs from at least two independent experiments are shown. Statistical significance was determined by two-tailed Student’s t-test. ***, *P* < 0.001. (B) Transcriptional *var* gene profile at 12 hpi, as assessed by RNA sequencing, for CSA-panned parasites, control gRNA clone D11, and two GC-rich ncRNA knockdown clones (CSA-panned clones transfected with CRISPRi D10 and J2). The means ± SEMs from at least two independent experiments are shown. Download FIG S5, PDF file, 0.04 MB. Copyright © 2020 Barcons-Simon et al. 2020 Barcons-Simon et al. This content is distributed under the terms of the Creative Commons Attribution 4.0 International license.

10.1128/mBio.03054-19.8 TABLE S1 Differential gene expression in the CRISPRi lines compared to the control lines. Results are from EdgeR differential gene expression analysis at 12 and 24 hpi. Download Table S1, XLSX file, 0.1 MB. Copyright © 2020 Barcons-Simon et al. 2020 Barcons-Simon et al. This content is distributed under the terms of the Creative Commons Attribution 4.0 International license.

10.1128/mBio.03054-19.9 TABLE S2 dCas9 ChIP-seq results in CRISPRi and control lines. Genes with peaks in dCas9 ChIP, as obtained by MACS2 software analysis, are shown. Download Table S2, XLSX file, 0.02 MB. Copyright © 2020 Barcons-Simon et al. 2020 Barcons-Simon et al. This content is distributed under the terms of the Creative Commons Attribution 4.0 International license.

Given that the GC-rich gene family is located within several central chromosome regions that are silenced by facultative heterochromatin enriched in P. falciparum heterochromatin protein 1 (PfHP1), we hypothesized that HP1 occupancy would determine the variegated transcription profile for the GC-rich genes. Indeed, ChIP-seq using anti-HP1 antibodies revealed that, as with the *var* genes, all GC-rich genes except the single active GC-rich gene, which is adjacent to the active *var* gene, were enriched for HP1 (Fig. S6). Notably, in the CRISPRi clones, all GC-rich genes were enriched in HP1 (Fig. S6).

10.1128/mBio.03054-19.6 FIG S6 HP1 profile for GC-rich genes. (A) ChIP sequencing data show similar enrichment profiles of HP1 for the GC-rich ncRNA knockdown line (CRISPRi clone A11) and the control gRNA line (control clone D11). Coverage of the fold enrichment over the input amount for dCas9 computed with MACS2 software is represented in red for the CRISPRi clone, in green for the control gRNA clone D11, and in blue for the WT. Data are representative of two independent experiments at 12 hpi. (B) GC-rich ncRNA members show variegated HP1 enrichment corresponding to transcriptomic data. In control clone D11, only the active GC-rich gene PF3D7_0712100 (highlighted in green), adjacent to the active *var* (PF3D7_0712000; Fig. 3A), was not enriched by HP1, while all downregulated GC-rich genes in the CRISPRi A11 clone presented with HP1 enrichment. Download FIG S6, PDF file, 0.2 MB. Copyright © 2020 Barcons-Simon et al. 2020 Barcons-Simon et al. This content is distributed under the terms of the Creative Commons Attribution 4.0 International license.

### GC-rich ncRNA CRISPRi affects other multigene families encoding variant surface antigens.

**FIG 4:**
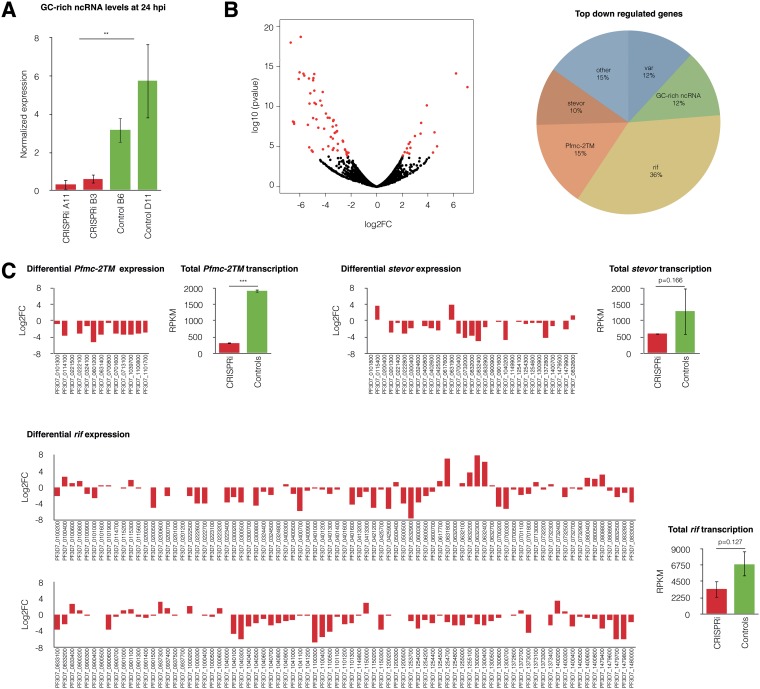
GC-rich ncRNA knockdown lines exhibit downregulation of the 2TM-type supergene family. (A) GC-rich ncRNA levels at 24 hpi, as quantified by RT-qPCR, for two CRISPRi clones (B3 and A11) and two control gRNA clones (control D11 and B6). The levels of transcription were normalized to housekeeping gene fructose-bisphosphate aldolase (PF3D7_1444800) transcription levels. The means ± SEMs from three independent experiments are shown. Statistical significance was determined by two-tailed Student's *t* test. **, *P* < 0.01. (B) (Left) Volcano plot for differential expression between CRISPRi clones and the scrambled control line. Differentially expressed genes with a 0.01 FDR cutoff are represented by red dots. The total number of significantly differentially expressed genes was 77, 77% of which were downregulated and 23% of which were upregulated. (Right) Families of top downregulated genes in CRISPRi clones significantly differentially expressed with a 0.01 FDR cutoff compared to their expression in scrambled control clones in three independent replicates. (C) (Left) Differential expression of 2TM multigene families for CRISPRi clones compared to that for the scrambled control line shows a general downregulation of most *Pfmc-2TM*, *stevor*, and *rif* gene family members. The log of the fold enrichment (log_2_ fold change [log2FC]) for three replicates of two CRISPRi clones compared to the gene expression in two scrambled control clones is represented. (Right) Mean ± SEM transcriptional levels of entire gene families, as assessed by RNA-seq, for three replicates of two CRISPRi and two scrambled control clones. Statistical significance was determined by two-tailed Student’s *t* test. ***, *P* < 0.001; **, *P* < 0.01.

GC-rich ncRNA is highly transcribed at the same later time point (∼24 hpi) as the *rif* and *stevor* genes (21, 22). Interestingly, several virulence gene families with a transcriptional peak in the blood-stage cycle later than that for *var* genes showed significant transcriptional downregulation upon GC-rich gene CRISPRi, even at 12 hpi (Fig. 3B and Table S1). To investigate a potential role for GC-rich ncRNA in the transcriptional control of these gene families, we performed RNA-seq and differential expression analysis in the CRISPRi lines at 24 hpi. The total number of genes significantly differentially expressed (FDR cutoff, 0.01) between the CRISPRi clones and the scrambled control clones at 24 hpi was 77, of which the majority (77%) were downregulated (Fig. 4B, Fig. S7, and Table S1) in three independent replicates. Besides GC-rich genes, most significantly downregulated genes belonged to multigene families encoding variant surface antigens with 2 transmembrane (2TM) domains (23): *rif*, *Pfmc-2TM*, and *stevor* (Fig. 4B). These three multigene families exhibited a transcription downregulation of most members in the CRISPRi lines compared to their expression in the control lines (Fig. 4C). In the case of the *Pfmc-2TM* gene family, the global transcription level was significantly lower in the CRISPRi lines than in the control lines, whereas the total levels of the *stevor* and *rif* gene families were not significantly lower. Altogether, these data strongly suggest that the GC-rich ncRNA is an important *trans*-activating element shared by at least the *Pfmc-2TM* and *var* gene families.

10.1128/mBio.03054-19.7 FIG S7 Differential gene expression in GC-rich ncRNA knockdown at 24 hpi. (A) Mean-variance scatterplot for differential expression between CRISPRi clones (A11 and B3) and scrambled control clones (D11 and B6) for three independent replicates at 24 hpi. (B) Volcano plot for differential expression between CRISPRi clones (A11 and B3) and scrambled control clones (D11 and B6) for three independent replicates at 24 hpi. Differentially expressed genes with a 0.01 FDR cutoff are represented by red dots. (C) Plot showing percentage of up- and downregulated genes amongst the differentially expressed genes with a 0.01 FDR cutoff in CRISPRi clones (A11 and B3) compared to scrambled control clones (D11 and B6) for three independent replicates at 24 hpi. (D) Families of top downregulated genes in CRISPRi clones (A11 and B3) compared to scrambled control clones (D11 and B6), significantly differentially expressed with a 0.01 FDR cutoff in three independent replicates at 24 hpi. Download FIG S7, PDF file, 1.2 MB. Copyright © 2020 Barcons-Simon et al. 2020 Barcons-Simon et al. This content is distributed under the terms of the Creative Commons Attribution 4.0 International license.

## DISCUSSION

The perinuclear compartment that is key to the mutually exclusive expression of a single *var* gene in P. falciparum remains poorly understood. In a previous study, we identified the GC-rich gene family to be the first *trans*-acting ncRNA localizing to this expression site (18). Here, we show that this ncRNA is essential for the transcriptional activation of a single *var* gene, and we provide evidence that this function of the GC-rich element is shared with other clonally variant gene families.

By performing RNA-seq analysis on freshly cloned parasite lines that each transcribed a single *var* gene, we showed that GC-rich ncRNAs are transcribed in a clonally variant manner (Fig. 1B). We observed two profiles of GC-rich gene transcription, depending on the relative chromosomal location of the GC-rich genes and active *var* gene. In cases in which there was one GC-rich gene predominantly transcribed (transcribed at a level 5- to 10-fold higher than that for other members), it was always found adjacent to the 5′ region of an active central *var* gene. The ncRNA transcription profile for a clone with an active central *var* gene or a subtelomeric *var* lacking an adjacent member of the GC-rich gene family at its 5′ upstream region showed multiple ncRNA transcripts, but at levels much lower than those in the former case. It is tempting to speculate that high levels of GC-rich ncRNA transcription adjacent to a central *var* gene may stabilize the expression site of a central *var* gene over that of subtelomeric *var* genes. A previous study reported variable switch rates, depending on the chromosomal location of *var* genes, with central *var* genes being more stably expressed and less prone to switching than the subtelomeric ones (24). Our data suggest that varying levels of ncRNA at the *var* gene expression site modulate the switch rate of individual *var* genes. Furthermore, transcription of GC-rich genes may open the local chromatin structure and enhance the accessibility of the transcription machinery to the adjacent *var* gene. This hypothesis finds support from the findings of a recent study showing the increased chromatin accessibility of GC-rich genes when they are adjacent to the active *var* gene and/or *rif* gene (25).

Until recently, it was not possible to inactivate an entire multigene family dispersed over many chromosomes in P. falciparum. We adapted the CRISPRi technique for the simultaneous knockdown of the entire GC-rich multigene family by targeting a conserved region that includes part of the polymerase III (Pol III) B box, present in all 15 members. All members of this GC-rich family have unique DNA motifs (internal A and B boxes) (18, 19) found only at polymerase III-transcribed tRNA genes and short interspersed nuclear elements (SINEs) in other organisms (26), suggesting that transcription of this multigene family is mediated by Pol III. Upon downregulation of GC-rich ncRNA transcription, *var* gene expression was abolished, revealing an unprecedented regulatory interaction between Pol III- and Pol II-transcribed clonally variant genes.

Interestingly, the GC-rich gene family is conserved throughout all *Laverania* subgenera of *Plasmodium*, along with *var* genes and other clonally variant gene families involved in immune evasion, such as *rif* and *stevor* (27–29). Since GC-rich genes are transcribed at their highest levels at approximately 24 hpi, the question arises whether GC-rich ncRNA might also play a direct or indirect role in regulating clonally variant virulence gene families expressed at later stages of the asexual blood cycle, such as *rif* (21, 30). A previous study suggested that an activation factor may be common to multiple clonally variant families (31). Our work suggests that GC-rich ncRNA could indeed be such a factor regulating different clonally variant gene families.

Although the precise molecular mechanism of ncRNA action remains to be investigated using techniques such as chromatin isolation by RNA purification (ChIRP), we postulate that the ncRNA associates with *var* gene control regions and acts as an activator. Indeed, a recent study showed that Pol III-transcribed SINEs act as enhancers of Pol II gene activation in response to the depolarization of neurons (32). However, the lack of sequence homology between GC-rich genes and *var* loci suggests the need for additional protein factors for such a physical interaction. Alternatively, GC-rich ncRNA could interact with nascent *var* mRNA, stabilizing it for transcription. It is also possible that GC-rich ncRNA could participate in ncRNA-mediated HP1 eviction from heterochromatic *var* genes, as previously described in the fission yeast Schizosaccharomyces pombe (33). Whichever hypothesis is correct, an essential next step would be to use this ncRNA as a molecular tool to pull down interacting partners from the *var* expression site, elucidating the molecular mechanism of *var* gene activation.

In conclusion, we developed a novel CRISPRi system that allows for the simultaneous downregulation of the entire GC-rich multigene family. In doing so, we establish the GC-rich ncRNA as an epigenetic regulatory element that plays a role in the activation of *var* gene transcription and the transcription of several other clonally variant gene families. We also provide a first glimpse into the molecular process that controls the switch rates of *var* genes, which is currently a black box in the field of *var* gene transcription. The identification of a *trans*-activating factor of the expression site opens novel experimental approaches to identify regulatory proteins needed for mutually exclusive *var* expression.

## MATERIALS AND METHODS

### Parasite culture and synchronization.

Blood-stage P. falciparum parasites were cultured as previously described (12). Parasites were synchronized with a 6-h time window by sorbitol lysis during the ring stage, followed by plasma gel enrichment in the schizont stage and another sorbitol treatment 6 h afterward, corresponding to 3 ± 3 hpi. Synchronized parasites were harvested at a 3.3% hematocrit and ∼2 to 4% parasitemia. Parasite development was monitored by Giemsa staining.

### Receptor panning.

Plastic cell culture dishes were coated with 1 mg/ml CSA receptor (catalog number C9819; Sigma) in phosphate-buffered saline (PBS) overnight at 4°C. The dishes were blocked with 1% bovine serum albumin (catalog number A4503; Sigma) in PBS for 1 h at 37°C. Mature-stage infected red blood cells (iRBCs) were isolated by plasma gel enrichment and resuspended in 8 ml of binding medium (RPMI 1640, 25 mM HEPES, pH 7.2). The culture dish was washed with binding medium, and iRBCs were added and allowed to adhere for 1 h with gentle agitation. The dish was washed until no unbound cells were observed by microscopy and bound cells were recovered with the culture medium.

### RNA isolation and reverse transcription-quantitative PCR (RT-qPCR).

RNA was harvested from synchronized parasite cultures after saponin lysis in 0.075% saponin in PBS, followed by one wash in PBS and resuspension in the QIAzol reagent. Total RNA was extracted using an miRNeasy minikit and performing on-column DNase treatment (Qiagen). Reverse transcription was achieved using a SuperScript Vilo kit (Thermo Fisher Scientific) and random hexamer primers. cDNA levels were quantified by quantitative PCR in a CFX384 real-time PCR detection system (Bio-Rad) using Power SYBR green PCR master mix (Applied Biosystems) and primers from a previous study (18). Transcript levels were quantified by normalizing the starting quantity mean to the one for a housekeeping gene (fructose-bisphosphate aldolase, PF3D7_1444800). The starting quantity means from three replicates were extrapolated from a standard curve of serial dilutions of genomic DNA.

### Chromatin immunoprecipitation and next-generation sequencing.

ChIP was performed as previously described (12), with some modification, using ring- or-trophozoite stage parasites (12 or 24 hpi). Sonicated chromatin (500-ng DNA content) was combined with either 0.5 μg of anti-HP1 (Genscript) or 1 μg of anti-hemagglutinin (anti-HA; catalog number Ab9110; Abcam) polyclonal rabbit antibodies. After overnight incubation, 25 μl of Dynabeads protein G (Invitrogen) was added to each sample, and an additional incubation of 2 h was conducted. Subsequent washing, cross-link reversion, and DNA extraction were carried out as described before (12). Sequencing libraries were produced with the immunoprecipitated DNA using a MicroPlex library preparation kit (v2; Diagenode) with Kapa HiFi polymerase (Kapa Biosystems) for the PCR amplification. For each ChIP sample, a control DNA corresponding to the ChIP input was processed in parallel. The multiplexed libraries were subjected to 150-bp paired-end sequencing on a NextSeq 500 sequencer (Illumina). Fastq files were obtained by demultiplexing the data using bcl2fastq software (Illumina), prior to downstream analysis. A minimum of two biological replicates were analyzed for each clone and time point.

### RNA library preparation and sequencing.

Total RNA was subjected to rRNA depletion to ensure ncRNA and mRNA capture using a RiboCop rRNA depletion kit (Lexogen), prior to strand-specific RNA-seq library preparation using a TruSeq stranded RNA LT kit (Illumina) with Kapa HiFi polymerase (Kapa Biosystems) for the PCR amplification. The multiplexed libraries were subjected to 150-bp paired-end sequencing on a NextSeq 500 sequencer (Illumina). Fastq files were obtained by demultiplexing the data using bcl2fastq software (Illumina), prior to downstream analysis. A minimum of three biological replicates were analyzed for each clone for the knockdown experiment, and a minimum of two biological replicates were analyzed for each WT clone (see Table S3 in the supplemental material).

10.1128/mBio.03054-19.10 TABLE S3 RNA-seq results. The RPKM of *var* and GC-rich ncRNA in wild-type clones and the CRISPRi lines are shown. Download Table S3, XLSX file, 1.4 MB. Copyright © 2020 Barcons-Simon et al. 2020 Barcons-Simon et al. This content is distributed under the terms of the Creative Commons Attribution 4.0 International license.

### Plasmid construction, transfection, and plasmid removal.

A sequence coding for a control guide RNA (gRNA; targeting luciferase) flanked by an autocleavable ribozyme (custom gene synthesis; GenScript) was ligated with the backbone vector pL6-eGFP (34) using the DraII and SwaI restriction sites and T4 DNA ligation to obtain the pL8-gRNA-control plasmid. A sequence coding for a gRNA targeting all GC-rich ncRNA loci flanked by an autocleavable ribozyme (gBlock; Integrated DNA Technologies) was cloned into the pL8-gRNA-control plasmid using the HpaI and PmlI restriction sites, followed by Gibson assembly (In-Fusion HD cloning kit; Clontech) to generate the pL8-gRNA-GC-tc plasmid. The constructs were transformed into Escherichia coli XL10-Gold ultracompetent cells (Agilent Technologies) and isolated with a NucleoBond Xtra Maxiprep kit (Macherey-Nagel). pUF-dCas9-GFP-HA was constructed by primer mutagenesis of pUF1-Cas9 and addition of a green fluorescent protein (GFP) tag, three HA tags, and an inducible riboswitch (35). pUF-dCas9-GFP-HA was transfected together with either pL8-gRNA-control or pL8-gRNA-GC-tc into ring-stage parasites as previously described (36) and maintained under WR99210 and DSM1 drug selection pressure. Parasite clones were obtained by limiting dilution. Rescue lines were obtained by drug removal from the CRISPRi lines and negative-selection treatment over 21 days using 5-fluorocytosine (Ancotil) to ensure pL8-gRNA-GC-tc removal. Negative selection was further maintained during parasite cloning.

### ChIP-seq and RNA-seq data analysis.

Fastq files were subjected to quality control using FastQC software (37). Sequencing reads were mapped to the P. falciparum genome (8) (PlasmoDB, v9), using the Burrows-Wheeler alignment tool (BWA-MEM) with the default settings (38). PCR duplicates were removed without further quality score filtering since 4 GC-rich ncRNAs fall in low-mapability regions. ChIP-seq data were normalized over the input, and likelihood ratio calculation and peak calling were performed using MACS2 software (39) with the default parameters and a false discovery rate (FDR) cutoff of 0.05. RNA-seq data gene counts were calculated using the bedtools suite of utilities (40). Differential gene expression analysis was performed with the R package edgeR (41) with an FDR threshold of 0.01. Normalization of the gene counts according to the number of reads per kilobase per million mapped reads (RPKM) for gene length and sequencing depth was performed using the R:limma package (42). Data were visualized using the Integrative Genomics Viewer (43).

### Data availability.

Sequencing data from this study are available in the GenBank repository under accession number PRJNA498234 (https://www.ncbi.nlm.nih.gov/bioproject/PRJNA498234).
