# Supplementary figures and images for: CRISPR Interference of a Clonally Variant GC-Rich Noncoding RNA Family Leads to General Repression of var Genes in Plasmodium falciparum
Source: mBio. 2020 Jan 21;11(1):e03054-19. doi: 10.1128/mBio.03054-19 (PMC6974570; doi:10.1128/mBio.03054-19)

# FIGURE S1

## A

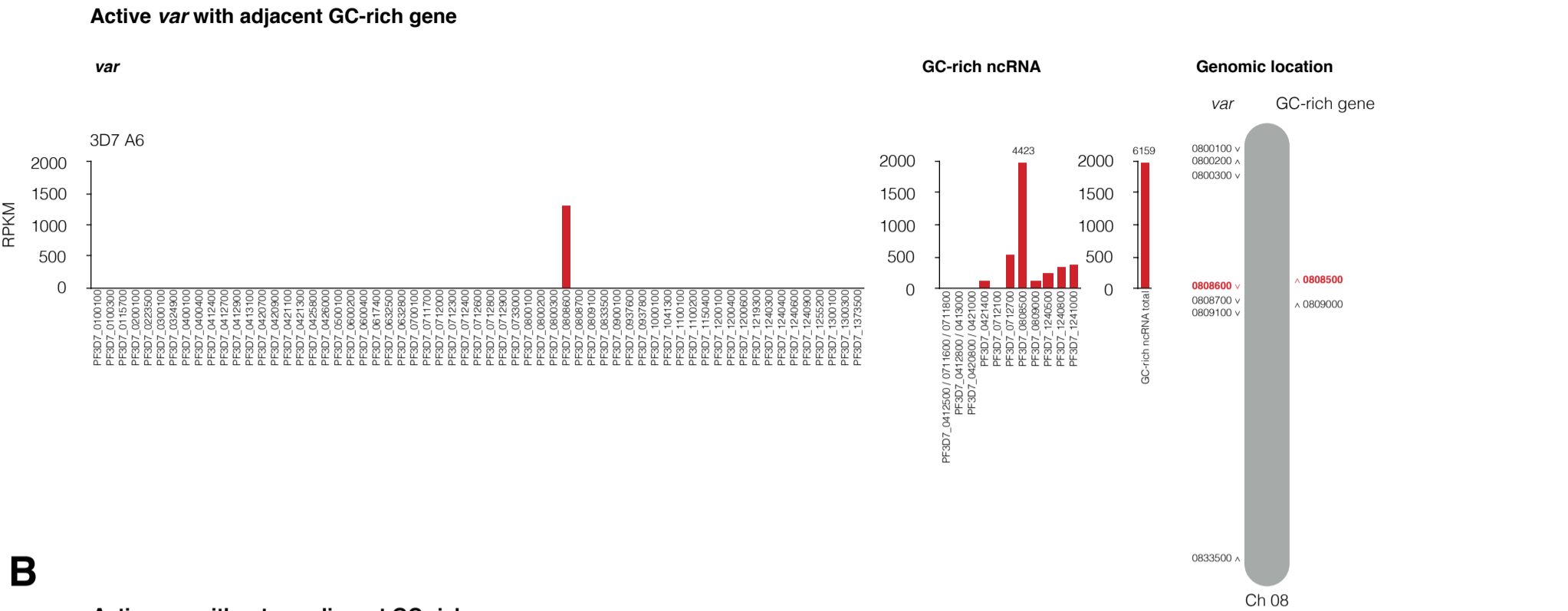

## B

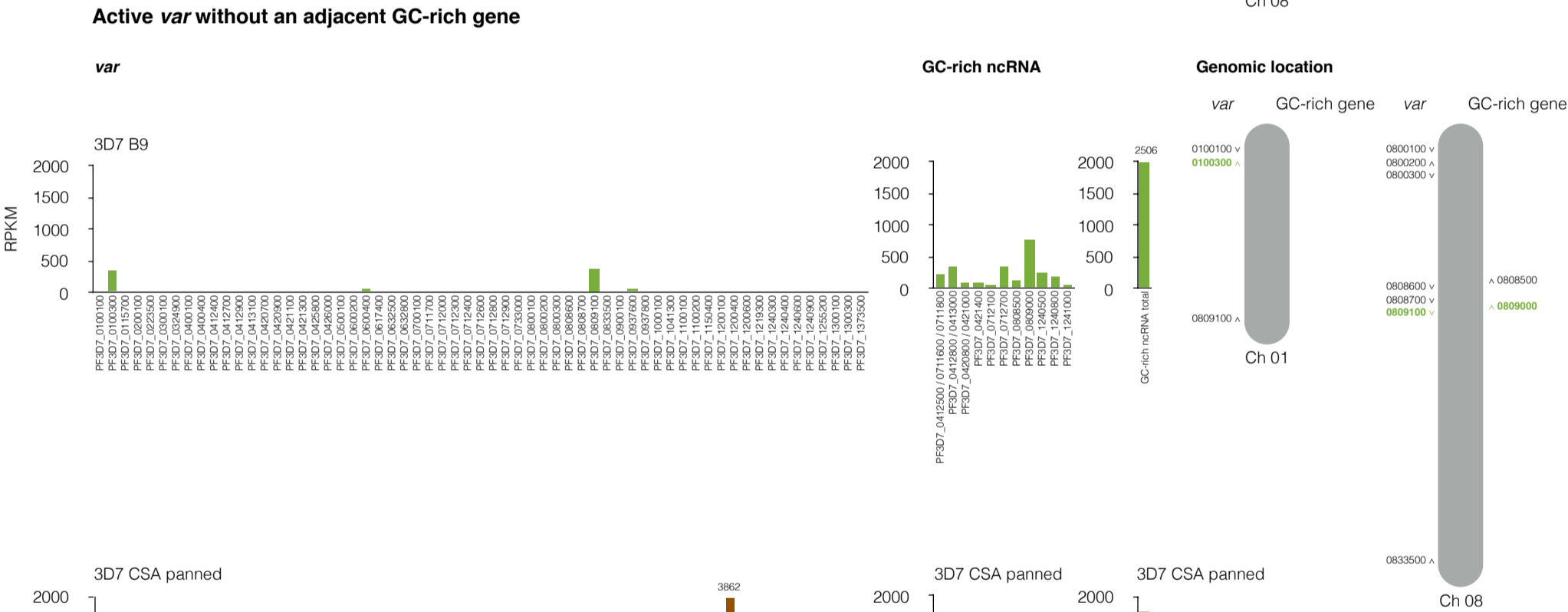

Supplement: FIG S1 [file mBio.03054-19-sf001.pdf]

FIGURE S2

A

dCas9 ChIP enrichment at GC-rich genes at 12hpi

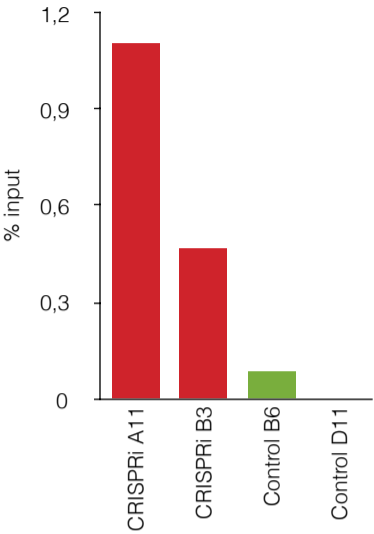

B

dCas9 ChIP at GC-rich genes at 24hpi

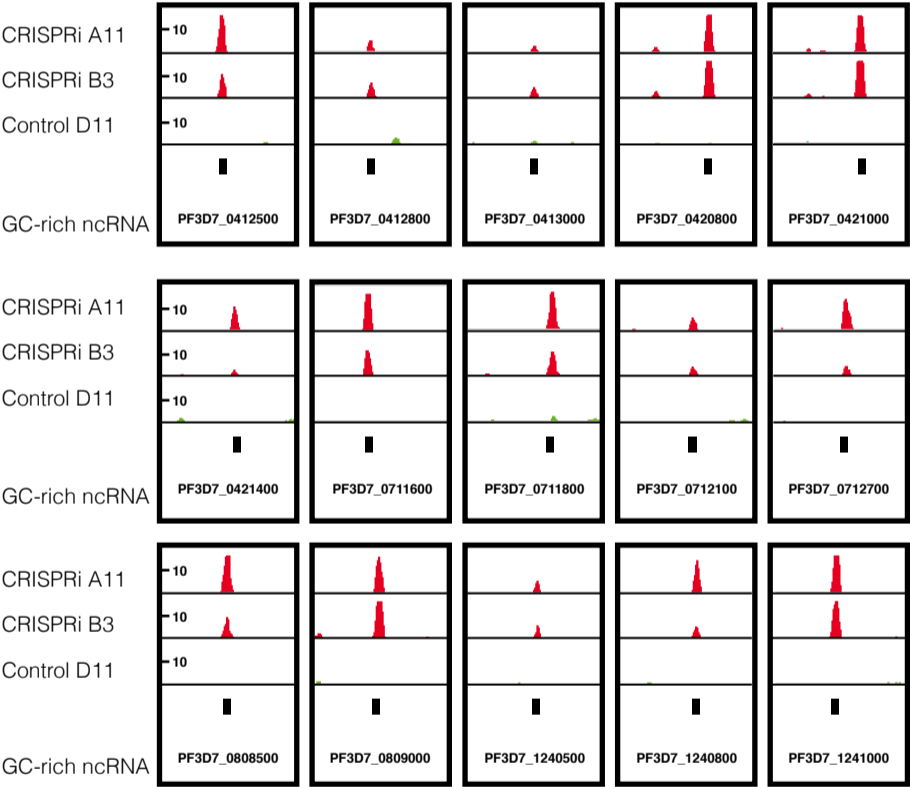

Supplement: FIG S2 [file mBio.03054-19-sf002.pdf]

**FIGURE S3**

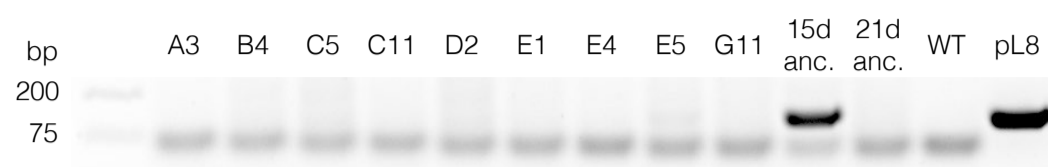

Supplement: FIG S3 [file mBio.03054-19-sf003.pdf]

FIGURE S5

A

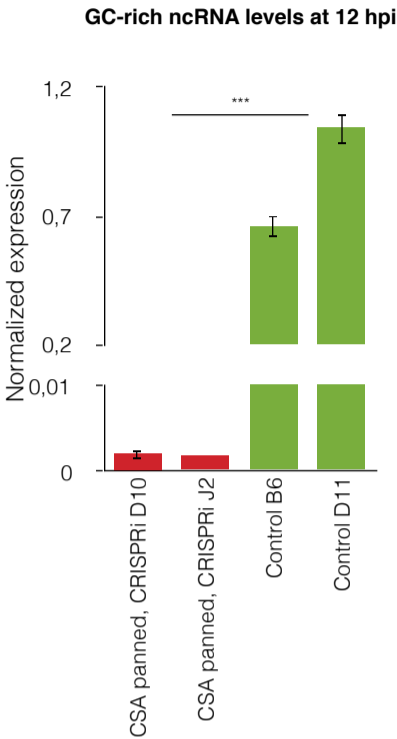

B

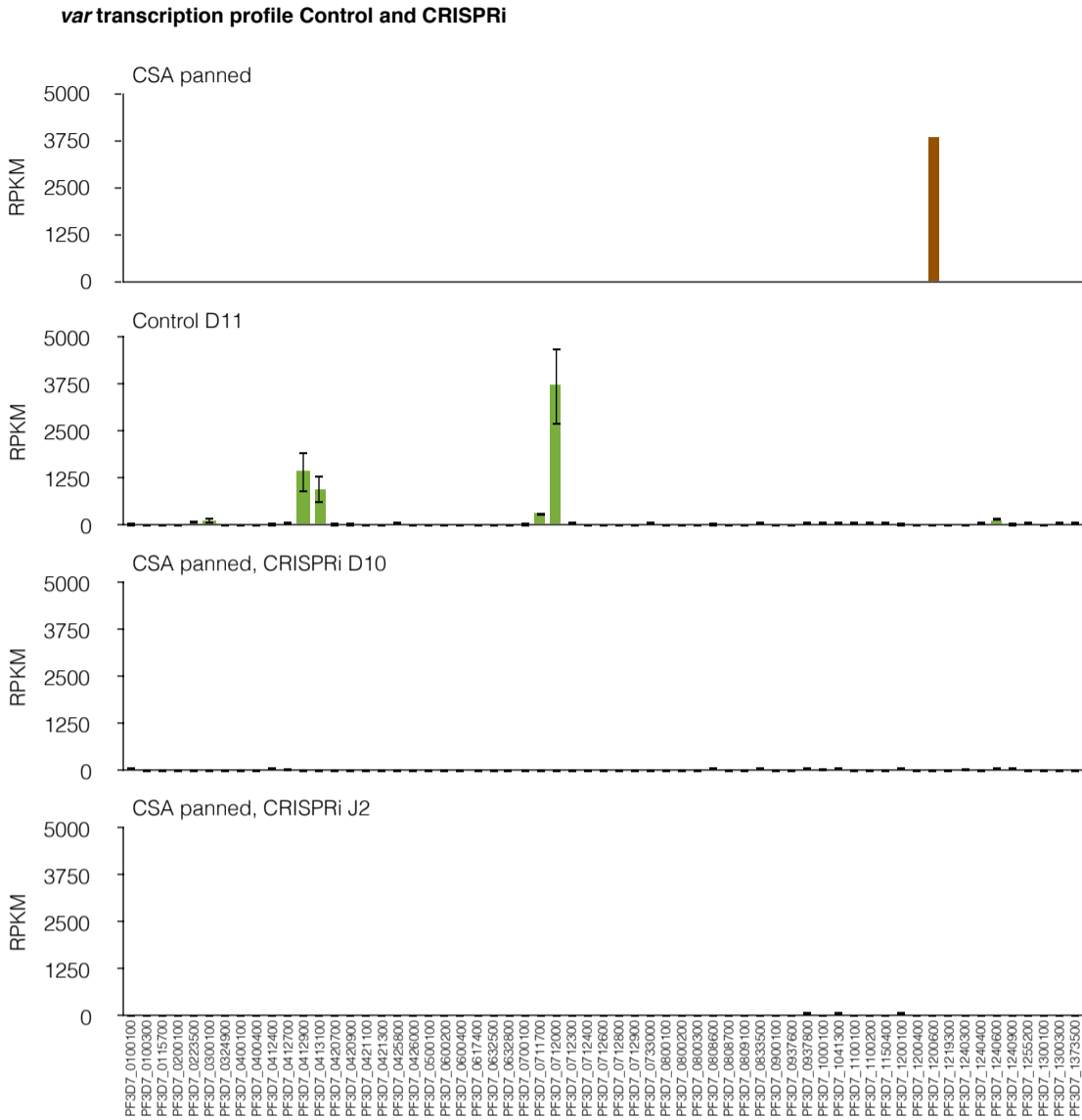

Supplement: FIG S5 [file mBio.03054-19-sf005.pdf]

FIGURE S5

A

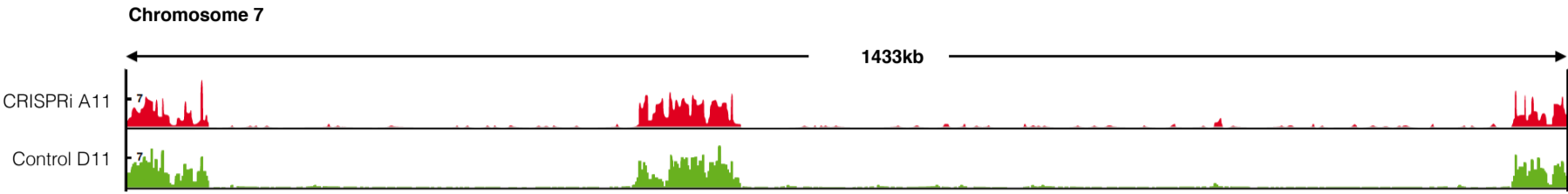

B

HP1 enrichment in GC-rich gene family

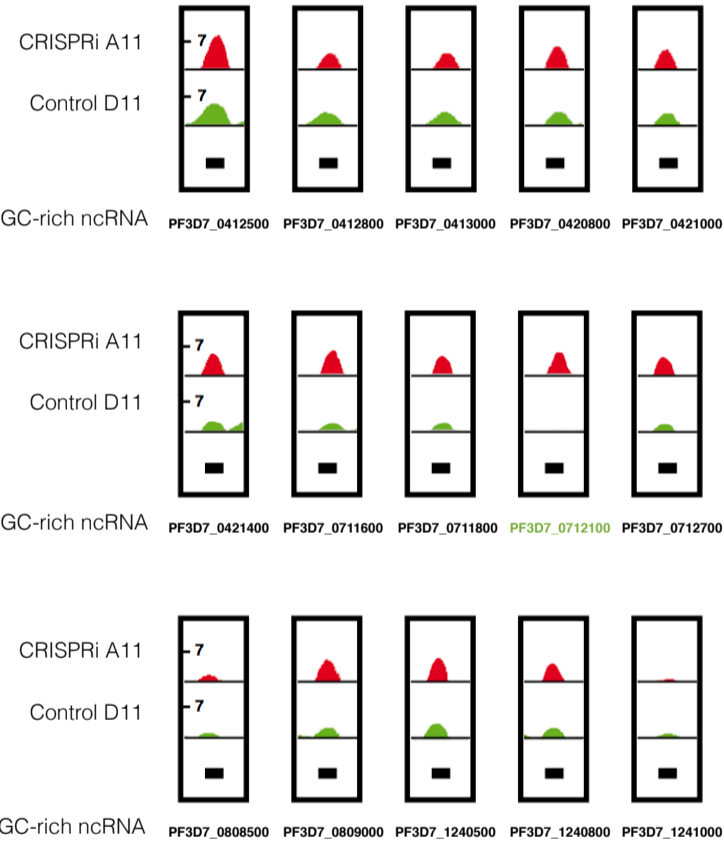

Supplement: FIG S6 [file mBio.03054-19-sf006.pdf]
