# Supplementary material for: CRISPR Interference of a Clonally Variant GC-Rich Noncoding RNA Family Leads to General Repression of var Genes in Plasmodium falciparum
Source: mBio. 2020 Jan 21;11(1):e03054-19. doi: 10.1128/mBio.03054-19 (PMC6974570; doi:10.1128/mBio.03054-19)
Supplement: FIG S4 [file mBio.03054-19-sf004.pdf]

FIGURE S4

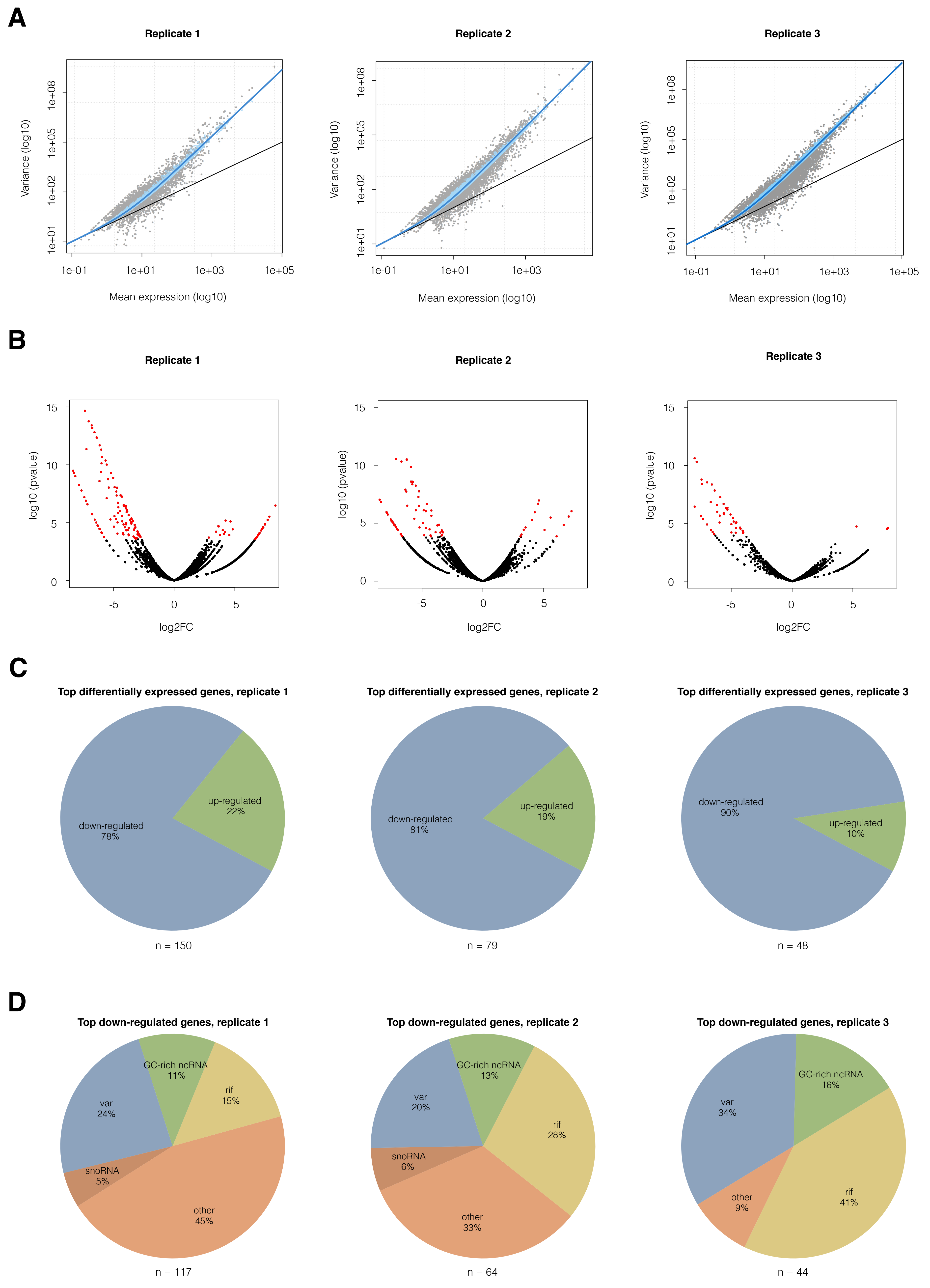

A
Replicate 1
Replicate 2
Replicate 3
Variance (log10)
1e+01
1e+02
1e+05
1e+08
1e-01
1e+01
1e+03
1e+05
Mean expression (log10)
B
Replicate 1
Replicate 2
Replicate 3
log10 (pvalue)
0
5
10
15
-5
0
5
log2FC
C
Top differentially expressed genes, replicate 1
down-regulated 78%
up-regulated 22%
n = 150
Top differentially expressed genes, replicate 2
down-regulated 81%
up-regulated 19%
n = 79
Top differentially expressed genes, replicate 3
down-regulated 90%
up-regulated 10%
n = 48
D
Top down-regulated genes, replicate 1
GC-rich ncRNA 11%
rif 15%
var 24%
snoRNA 5%
other 45%
n = 117
Top down-regulated genes, replicate 2
GC-rich ncRNA 13%
var 20%
rif 28%
snoRNA 6%
other 33%
n = 64
Top down-regulated genes, replicate 3
GC-rich ncRNA 16%
var 34%
other 9%
rif 41%
n = 44
