# Supplementary material for: CRISPR Interference of a Clonally Variant GC-Rich Noncoding RNA Family Leads to General Repression of var Genes in Plasmodium falciparum
Source: mBio. 2020 Jan 21;11(1):e03054-19. doi: 10.1128/mBio.03054-19 (PMC6974570; doi:10.1128/mBio.03054-19)
Supplement: FIG S7 [file mBio.03054-19-sf007.pdf]

FIGURE S7

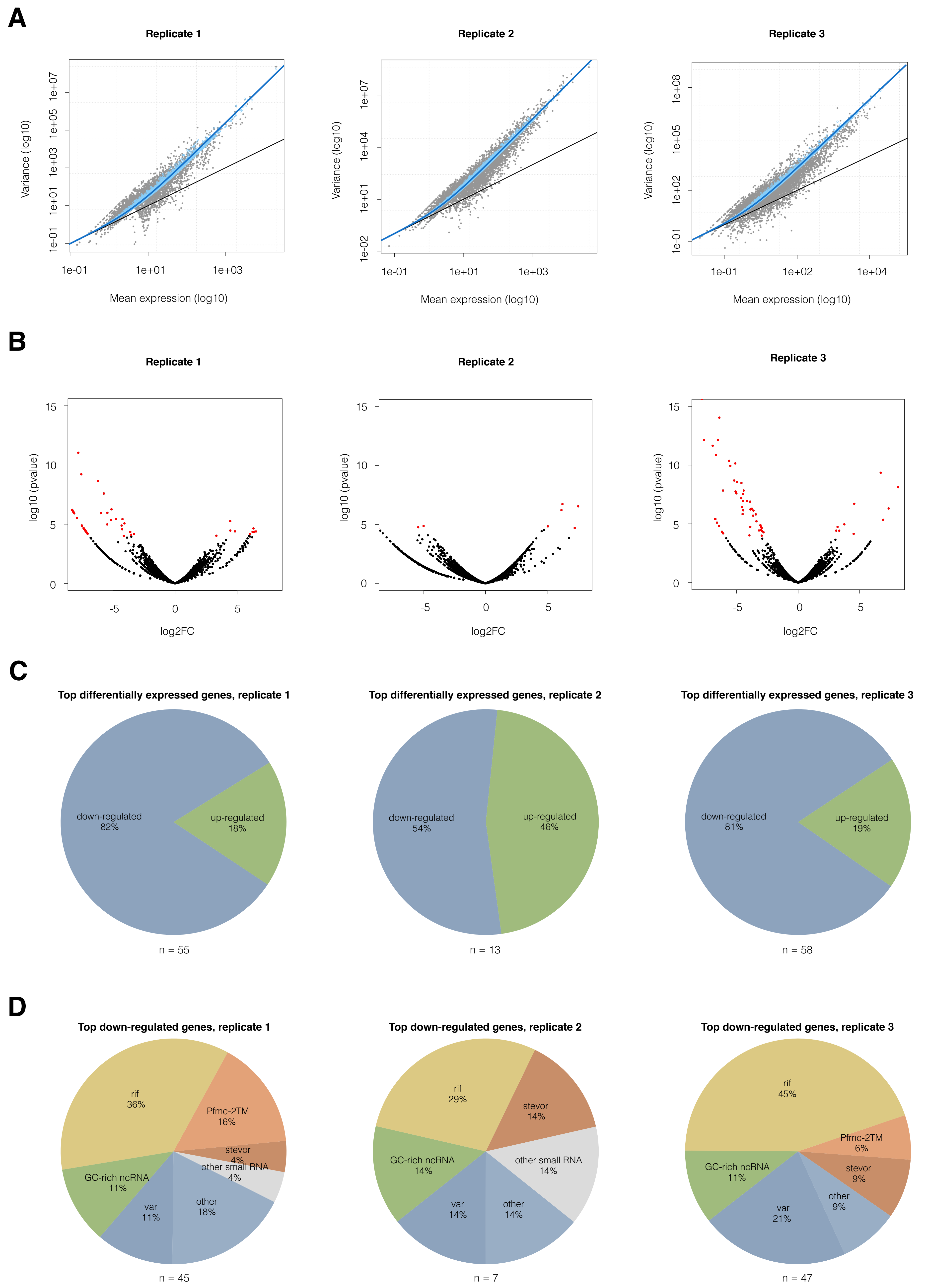
A
Replicate 1
Replicate 2
Replicate 3
Variance (log10)
Mean expression (log10)
1e-01
1e+01
1e+03
1e+05
1e+07
1e-02
1e+04
1e+02
1e+08
B
Replicate 1
Replicate 2
Replicate 3
log10 (pvalue)
log2FC
0
5
10
15
-5
C
Top differentially expressed genes, replicate 1
down-regulated 82%
up-regulated 18%
n = 55
Top differentially expressed genes, replicate 2
down-regulated 54%
up-regulated 46%
n = 13
Top differentially expressed genes, replicate 3
down-regulated 81%
up-regulated 19%
n = 58
D
Top down-regulated genes, replicate 1
rif 36%
Pfmc-2TM 16%
stevor 4%
other small RNA 4%
other 18%
var 11%
GC-rich ncRNA 11%
n = 45
Top down-regulated genes, replicate 2
rif 29%
stevor 14%
other small RNA 14%
other 14%
var 14%
GC-rich ncRNA 14%
n = 7
Top down-regulated genes, replicate 3
rif 45%
Pfmc-2TM 6%
stevor 9%
other 9%
var 21%
GC-rich ncRNA 11%
n = 47
